# Adulteration of Drugs

**Published:** 1888-08

**Authors:** 


### ADULTERATION OF DRUGS.

It is passing strange that there is no statute in the Code of Georgia providing punishment for the offense of adulterating drugs. It is a tribute to the honesty of our druggists that no need for such a law has been felt heretofore. A case has recently come before the City Court of Atlanta which justifies the medical profession and pharmacists in urging upon our Legislature the necessity of framing such a law.

In the case referred to, the druggist was charged with cheating and swindling, in that he had sold subnitrate of bismuth adulterated with a large amount of wheat flour.

Unfortunately the indictment was quashed, because of a flaw detected by the defendant's lawyers.

It is understood that the case will come up again in the fall.

It is not our purpose to pass upon the guilt or innocence of a man whose cause will be decided by due process of law. We cannot refrain, however, from saying a few earnest words suggested by this case.

In all the category of misdemeanors and crimes, it is impossible to conceive of a meaner offense than selling impure and adulterated drugs. Burglary in the night-time is a most commendable act compared with it, and picking a man's pocket becomes a positive virtue. To knowingly sell adulterated drugs implies a willingness to take away from a suffering fellow-creature the chance (it may be, the only one) of baffling death. Viewed from this standpoint, it amounts morally to homicide. We hope all the doctors and pharmacists of Georgia will urge upon their representatives in the Legislature the pressing need of providing a remedy against such an offense.

